# Neurological Presentation of Invasive Mucormycosis

**DOI:** 10.7759/cureus.28104

**Published:** 2022-08-17

**Authors:** Natalie Torrente, Amy Kiamos, Madeline Fasen

**Affiliations:** 1 Internal Medicine, University of Florida College of Medicine - Jacksonville, Jacksonville, USA

**Keywords:** mucormycosis, rhizopus, invasive fungal sinusitis, preseptal cellulitis, diabetes

## Abstract

An elderly female presented to the emergency department with a right-sided facial droop and headache for two weeks. Investigations revealed poorly controlled diabetes, and the patient was found to be in diabetic ketoacidosis. Maxillofacial computed tomography (CT) demonstrated right postseptal cellulitis with concern for acute invasive fungal sinusitis. The patient was taken to the operating room for orbital surgical exploration and antrostomy. Surgical pathology revealed broad hyphae consistent with* Rhizomucor* species, and the patient was diagnosed with mucormycosis. Because the patient was not clinically improving, further imaging was obtained, which showed a large right retroantral phlegmon extending into the cranial fossa and right cavernous sinus, and the patient subsequently underwent surgical debridement. The following postoperative day, the patient was stroke-alerted due to altered mental status and inability to follow commands. She was found to have a small embolic infarct. Due to the poor prognosis of the patient, she was discharged with hospice. Mucormycosis is more commonly found in immunocompromised patients, such as those with uncontrolled diabetes mellitus but very rarely does it involve the cranium. This disease process is very important to recognize early due to high morbidity and mortality rates and devastating outcomes.

## Introduction

Mucormycosis is a rare fungal infection that typically starts as acute sinusitis with headache and facial pain. As the infection spreads beyond the sinuses into the orbit and surrounding tissues, patients will develop the classic presentation of facial erythema, periorbital edema, and proptosis. A black eschar and ulceration can further develop from tissue necrosis. We report a case of an unusual presentation of mucormycosis. The patient presented with predominantly stroke-like symptoms. The case demonstrates how a rare infection can present with common neurological symptoms.

## Case presentation

An elderly Caucasian female with a past medical history of uncontrolled insulin-dependent diabetes mellitus, atrial fibrillation, and anemia presented with a new right-sided facial droop. Her symptoms were associated with headache, right upper extremity weakness, diplopia, and right-sided facial pain. Her family reported that she started having frequent falls at home and bowel/bladder incontinence over the past two weeks. The patient admitted to fatigue, weight loss, poor appetite, and right eye pain with surrounding edema. There was no history of fevers, chills, nasal congestion, rhinorrhea, nausea, vomiting, diarrhea, shortness of breath, abdominal pain, or dysuria.

The patient’s physical examination consisted of a right-sided facial droop with facial asymmetry. There was mild right eye proptosis with periorbital erythema and edema. Her right eye had a conjunctival injection, conjunctival chemosis, and pupillary constriction. Her extraocular movements were intact and painful on the right. There were no weakness or sensation changes appreciated. She had 2+ pitting edema and dusky skin color changes in her lower extremities.

**Figure 1:**
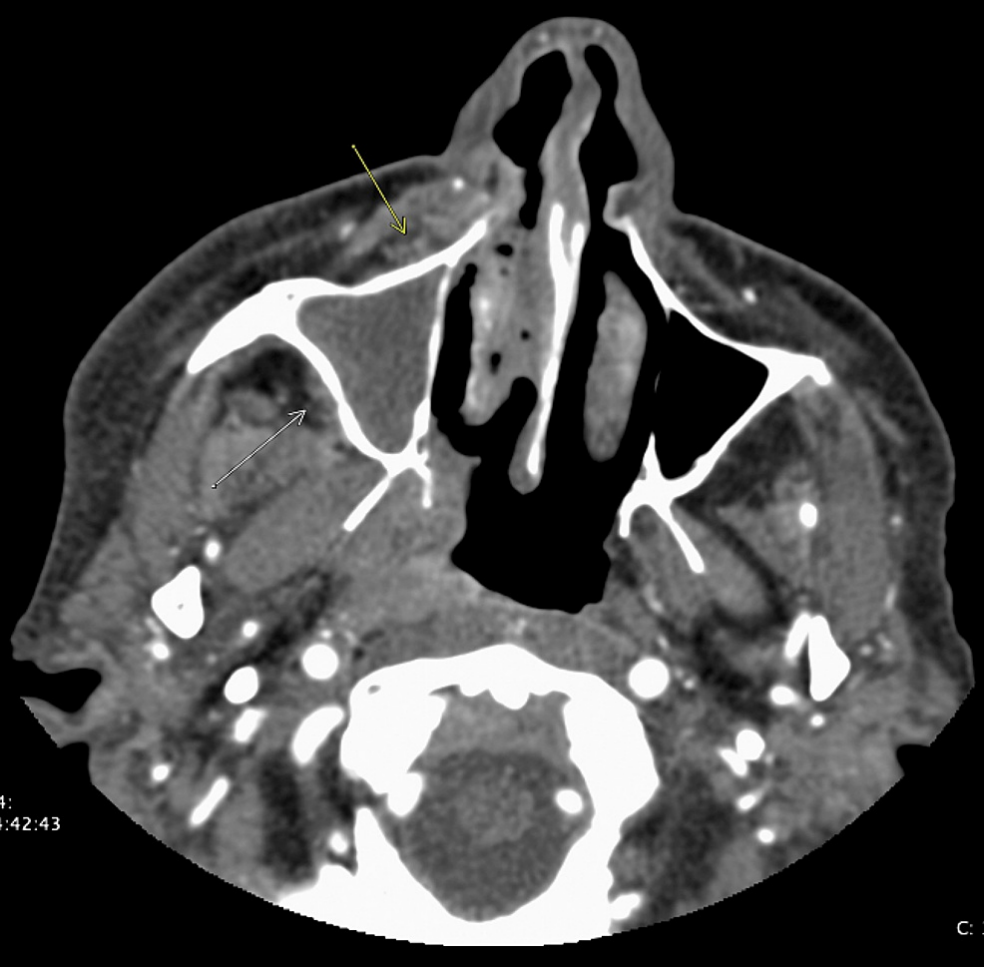
Right orbital infection with inflammatory changes in the right premaxillary (yellow arrow) and right retromaxillary fat (white arrow) concerning for invasive fungal sinusitis.

**Figure 2:**
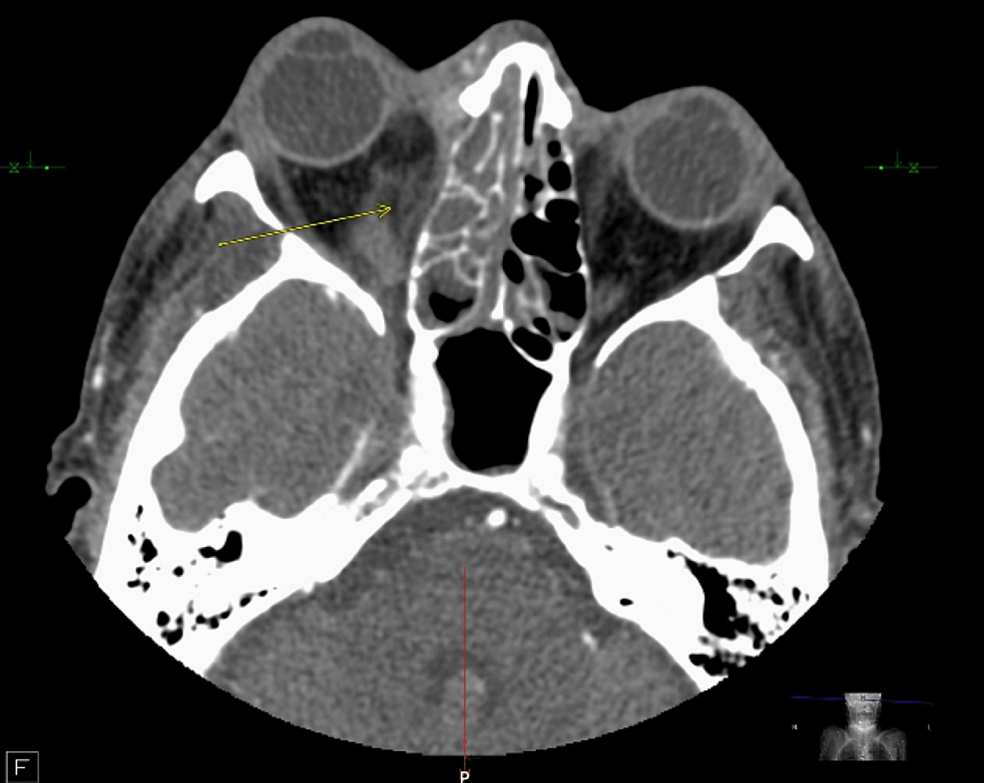
Right postseptal/orbital cellulitis with edema and inflammatory stranding in the inferior and medial right extraconal orbital space concerning for phlegmon (yellow arrow). Right proptosis is noted from mass effect. Severe opacification is noted in the right ethmoid air cells and right maxillary sinus.

Laboratory values demonstrated leukocytosis and hyperglycemia with evidence of diabetic ketoacidosis due to acidosis and elevated anion gap, as well as increased beta-hydroxybutyrate (Table 1). In addition, the patient has poorly controlled diabetes (Table 1). Maxillofacial computed tomography (CT) demonstrated right orbital infection with inflammatory changes concerning for invasive fungal cellulitis (Figure 1). In addition, there were inflammatory changes in the inferior and medial right extraconal orbital space concerning for phlegmon and right proptosis of the eye concerning for mass effect (Figure 2). Magnetic resonance imaging (MRI) was unable to be obtained initially due to the patient having a bladder stimulator that needed to be turned off for imaging.

**Table 1:** Important laboratory values on admission.

	Value	Reference
White blood cells	25.26	4.5-11 (thousand/mm^3^)
Glucose	418	71-99 mg/dL
Carbon dioxide total	14	21-29 mmol/L
Anion gap	24	4-16 mmol/L
Beta-hydroxybutyrate	46	0.2-2.8 mg/dL
C-reactive protein	262	<8 mg/L
Hemoglobin A1C	16.30%	4.8%-5.9%

She was diagnosed with invasive rhino-mucormycosis of the right maxillary sinus. Right orbital surgical exploration and antrostomy were performed. Surgical pathology of the right maxillary sinus and right orbital abscess demonstrated broad hyphae consistent with *Rhizomucor* species. Infectious disease recommended treatment with intravenous amphotericin, caspofungin, cefepime, and metronidazole. Her diabetic ketoacidosis resolved with insulin continuous drip and fluids. She initially started to improve with antibiotic and antifungal treatment.

After the bladder stimulator was turned off, she was able to get an MRI to determine the extent of the infection. She underwent another surgical debridement after MRI demonstrated a large right retroantral abscess extending into the cranial fossa and right cavernous sinus. The day after surgery, she was stroke-alerted due to an acute change in mentation. Repeat MRI at this time demonstrated multiple small embolic infarcts. Due to the severity of her disease and prognosis, she was discharged with hospice.

## Discussion

Mucormycosis is a rare fungal infection with high morbidity and mortality rates. The most common agents of mucormycosis are *Rhizopus* spp., *Mucor* spp., and *Lichtheimia* spp. [1]. Most cases of mucormycosis result from inhalation of fungal sporangiospores that have been released into the air or from direct inoculation onto the skin or gastrointestinal mucosa [2]. Until the past two decades, most published cases of mucormycosis had been in diabetic patients, but now, there are more cases in patients with impaired immune defenses including malignancies and hematopoietic stem cell transplants [2].

The primary mode of infection is through the inhalation of fungal spores. Most *Mucor* species form spores that are sufficiently small to reach the distal alveolar spaces (3-11 µm), whereas larger spores (>10 µm) can lodge into the nasal turbinates. However, in order to establish an infection, fungal spores have to overcome phagocytosis by macrophages and neutrophils and grow into hyphae, which is why mucormycosis rarely affects immunocompetent patients [2].

In a patient with diabetic ketoacidosis, there is a high incidence of mucormycosis caused by *Rhizopus oryzae*, also known as *Rhizopus arrhizus*, because they produce enzyme ketoreductase, which allows them to utilize the patient’s ketone bodies. Hyperglycemia can also stimulate fungal growth, and diabetes-associated reduction chemotaxis and phagocytic efficiency allow these organisms to proliferate [3].

Rhinocerebral mucormycosis is a rare but fatal infection of the nasal cavity and sinuses. It is known to exist in two forms: the well-known acute form and the less common chronic form. Acute rhinocerebral mucormycosis is an opportunistic but fulminant fungal infection of the nose, sinuses, orbit, and cranial structures. It can spread to the orbits and cranium within days [4]. In advanced disease, chemosis, ptosis, proptosis, ophthalmoplegia, and blindness and multiple cranial nerve palsies can result.

Radiographic imaging is helpful in establishing the extent of sinus, orbital, or intracranial progression of mucormycosis and in determining the efficacy of treatment. Early diagnosis and treatment are of extreme importance for the successful eradication of infection. The confirmation of the diagnosis of *Mucor* is best obtained with a tissue specimen from the junction of necrotic and non-necrotic tissue [5]. Medical management alone is not effective due to poor drug delivery to the infection site due to extensive vascular thrombosis [6]. Systemic antifungal therapy includes the use of high-dose amphotericin B and is associated with a survival rate of 72%. In addition, aggressive control of the underlying disease and debridement are needed [3].

Systemic antifungal chemotherapy, limited to amphotericin B, has significantly improved the survival rates of patients with mucormycosis [7]. This drug remains the only proven and effective medical therapy. Cessation of treatment is based on clinical evidence and the absence of disease [7]. Surgical procedures are also important in conjunction with antifungals. Drainage and debridement of paranasal sinuses, exenteration of necrotic orbital contents, palatectomy, and craniotomy have been associated with cure [8]. According to Blitzer et al., radical resection enhances survival from 57.5% to 78% in diabetic patients [8]. In addition, survival rates are low once a fungal abscess has formed [9]. Nussbaum and Hall reported that seven individuals treated for intracranial disease died in contrast to only four patients where *Mucor* was isolated to just the sinuses and orbits [10].

## Conclusions

This is an unusual presentation of mucormycosis infection presenting with predominantly stroke-like symptoms (right-sided facial droop, headache, and diplopia). Uncontrolled diabetes is an important risk factor for strokes and invasive mucormycosis. It is important to consider infectious causes of neurological presenting symptoms. It is vital to recognize mucormycosis infection early to prevent the spread of the infection deep into the orbits and cranium. Early initiation of antifungals and surgical debridement is essential to improve survival and prevent progression to devastating outcomes.

## References

[1] Petrikkos G, Skiada A, Lortholary O, Roilides E, Walsh TJ, Kontoyiannis DP (2012). Epidemiology and clinical manifestations of mucormycosis. Clin Infect Dis.

[2] Farmakiotis D, Kontoyiannis DP (2016). Mucormycoses. Infect Dis Clin North Am.

[3] Marx RE, Stern D (2006). Oral and maxillofacial pathology: a rationale for diagnosis and treatment. 1.

[4] Sachdeva K (2013). Rhino-oculo cerebral mucormycosis with multiple cranial nerve palsy in diabetic patient: review of six cases. Indian J Otolaryngol Head Neck Surg.

[5] Singh NP, Garg S, Kumar S, Gulati S (2006). Multiple cranial nerve palsies associated with type 2 diabetes mellitus. Singapore Med J.

[6] Smith JL, Stevens DA (1986). Survival in cerebro-rhino-orbital zygomycosis and cavernous sinus thrombosis with combined therapy. South Med J.

[7] Abramson E, Wilson D, Arky RA (1967). Rhinocerebral phycomycosis in association with diabetic ketoacidosis. Report of two cases and a review of clinical and experimental experience with amphotericin B therapy. Ann Intern Med.

[8] Blitzer A, Lawson W, Meyers BR, Biller HF (1980). Patient survival factors in paranasal sinus mucormycosis. Laryngoscope.

[9] Couch L, Theilen F, Mader JT (1988). Rhinocerebral mucormycosis with cerebral extension successfully treated with adjunctive hyperbaric oxygen therapy. Arch Otolaryngol Head Neck Surg.

[10] Nussbaum ES, Hall WA (1994). Rhinocerebral mucormycosis: changing patterns of disease. Surg Neurol.

